# Role of QRS fragmentation in 12-lead surface ECG in prediction of isolated epicardial coronary artery ectasia and its anatomical distribution in patients without acute coronary syndromes

**DOI:** 10.1186/s43044-020-00054-4

**Published:** 2020-04-15

**Authors:** Diaa Kamal, Moataz Hashem

**Affiliations:** 1 Cardiology Department, Faculty of Medicine, Ain Shams University, Abbasia Street, Cairo, Egypt; 2 Damietta Cardiology Center, Damietta, Egypt

**Keywords:** 12-lead surface ECG, QRS fragmentation, Coronary ectasia, Coronary angiography

## Abstract

**Background:**

Coronary artery ectasia (CAE) is a form of abnormal coronary artery lumen dilatation associated with epicardial flow disturbances and microvascular dysfunction. QRS complex fragmentation (fQRS) in surface ECG is caused by abnormal depolarization due to myocardial ischemia and scarring. It has been proved in different studies to be positively correlated with adverse cardiac events. This study aimed to assess the role of fQRS as a non-invasive predictor of CAE and its anatomical distribution. A total of 100 patients referred for elective coronary angiography were included and divided into 2 groups: 50 patients with isolated CAE (group A) and 50 patients with angiographically normal coronaries (group B, control group). Both groups were compared regarding clinical, echocardiographic, and ECG characteristics.

**Results:**

Univariate analysis showed a significant correlation between male sex, smoking, diabetes mellitus, increased systolic blood pressure, fQRS, echocardiographic evidence of diastolic dysfunction, and CAE (*P* values of 0.005, 0.002, 0.016, 0.027, 0.0001, and 0.04, respectively). Multivariate regression analysis showed that fQRS is the most important independent predictor for the presence of CAE (*P* < 0.00001) with sensitivity 94%, specificity 88%, PPV 88.7%, and NPV 93.6%. We also found a significant correlation between fQRS distribution in surface ECG and anatomical distribution of CAE [increased territories with multivessel affection (*P* = 0.00001), anterior leads with LAD affection (*P* = 0.00001), lateral and inferior leads with LCX affection (*P* = 0.003 and 0.04, respectively), inferior leads with RCA affection (*P* = 0.00001)].

**Conclusion:**

fQRS in surface ECG can potentially be used as an effective non-invasive method to predict isolated CAE and its anatomical distribution.

## Background

Coronary artery ectasia (CAE) is defined as an abnormal coronary dilatation (segmental or diffuse) > 1.5 times the reference normal vessel either in the same artery or in other adjacent normal arteries [1]. Markis et al. classified CAE into 4 types (types 1, 2, and 3 for diffuse ectasia and type 4 for focal ectasia) [2]. Historically, both terms (ectasia and aneurysm) were interchangeably used to address coronary aneurysmal dilatation [3]. Recently, authors confined the “aneurysm” term for focal coronary dilatation leaving the “ectasia” term for more diffuse coronary aneurysmal dilatation [4].

CAE is seen in approximately 5% of cases undergoing coronary angiography (CA) for various indications [3]. The exact etiology of CAE is not fully understood. Atherosclerosis, iatrogenic injury, vasculitis, and genetic factors might have a causal role [5–7]. Despite being mostly asymptomatic, local thrombosis in ectatic coronaries with or without distal embolization can lead to acute ischemic events [3]. It is known that CAE disturbs flow in coronary arteries increasing blood viscosity which consequently enhances tendency towards thrombosis [8]. Stress-induced ischemia without obstructive CAD due to microvascular dysfunction have been documented in patients with CAE, an abnormality commonly referred to as (dilated coronopathy) [9].

Long-term cardiovascular outcomes of patients with CAE suffering from acute coronary syndromes were always a question needing an answer. Doi et al. in their study showed a significant prognostic effect of CAE in these patients with improved outcomes in cases treated with therapeutic oral anticoagulation [10].

Fragmentation of QRS complex (fQRS) is an indicator of unsynchronized depolarization of myocardial fibers that may be attributed to myocardial ischemia or scarring that may even be subclinical [11]. Autopsy studies showed that a mixture of viable myocardium, necrotic myocardial fibers, and fibrosis is responsible for this finding in surface electrocardiogram (ECG) [12]. fQRS was repeatedly studied as an important prognosticator in different cardiac disorders [13–22].

Given the clinical consequences of CAE especially if it leads to an acute coronary syndrome, many studies were done trying to find predictors for this coronary abnormality [23–26]. fQRS despite being a very cheap and safe non-invasive method was not sufficiently studied as a potential predictor for isolated CAE. In our study, we tried to find if there is a significant correlation between isolated CAE and fQRS or not.

## Methods

Our study was a prospective observational case control study that included 100 patients presented to our Center in the time interval from November 2016 to September 2017.

The study was approved by the Research Ethics Committee of our institute, and all patients signed an informed written consent for participation in the study in accordance with the Declaration of Helsinki. The study included stable patients indicated for elective CA [patients with chest pain and high pretest probability for CAD that have not done any non-invasive testing or those who proved to have high-risk findings in non-invasive testing (stress ECG, radioisotope scanning, stress echo, etc.)] according to the appropriateness criteria [27]. Patients were divided according to CA data into 2 groups: isolated CAE group (50 patients) and control group which consisted of 50 patients with angiographically normal coronaries.

### Exclusion criteria

Patients with previous MI, pathological Q waves on ECG, QRS duration > 120 ms, paced rhythm, any type of cardiomyopathy, renal impairment (serum creatinine > 2 mg/dl), left ventricular hypertrophy (LVH) by ECG or by echocardiography, intake of anti-arrhythmic medications, and those who refused to sign the informed consent were excluded from the study.

All patients were subjected to proper history taking to obtain all clinical data regarding their demographic data, classic cardiovascular risk factors, and any previous medical history. Clinical examination was done including measuring arterial blood pressure (ABP). Transthoracic echocardiography was performed using a high-performance cardiovascular ultrasound system (Vivid S5 machine, GE healthcare Company, USA) with registration of various data.

### ECG acquisition and interpretation

**Fig. 1:**
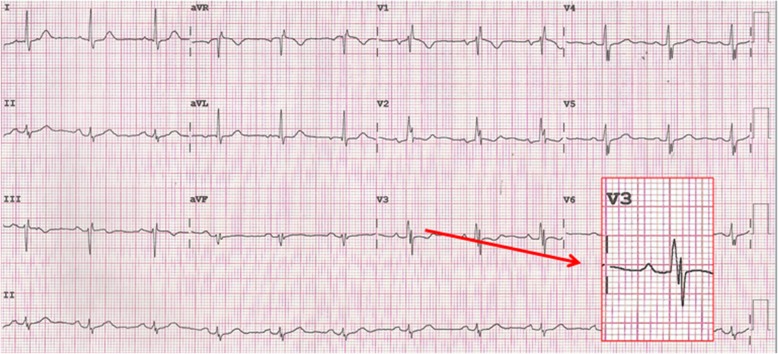
Twelve-lead surface ECG recording for one of our study population showing different patterns of QRS fragmentation in inferior, anterior, and lateral leads (R’ in V_1_, notched R wave in V_2_, notched S wave in II and V_4–6_). This patient was discovered to have CAE in LAD, LCX, and RCA by invasive CA. A magnified view of lead V_3_ is shown with 2 positive and 2 negative waves with a normal QRS complex duration

A 12-lead ECG machine (PageWriter TC20, Philips Medical Systems, USA) was used with the following parameters in recording: filter 0.16–100 Hz, AC filter 60 Hz, and speed 10 mm/mv and 25 mm/s. fQRS was considered to be present if any of the different RSR’ patterns was observed (additional R wave which is called R’, more than one R’ without a typical bundle branch block, notching of R or S waves) in 2 or more contiguous leads belonging to the same lead set representing territory of supply of a major coronary artery [28]. Anteroseptal leads were defined as leads V_1_–V_2_, anterior leads as leads V_1_–V_4_, anterolateral leads as leads V_1_–V_6_, high lateral leads as leads I and aVL, lateral leads as leads V_4_–V_6_, and inferior leads as leads II, III, and aVF. ECG analysis was done without magnification. Fragmentation was considered positive if seen in all complexes of the recorded ECG lead. The longest QRS was used to detect QRS duration in any lead. ECG recordings of all patients were revised by another cardiologist blinded to clinical and angiographic findings of patients. Figure 1 shows a diagrammatic illustration of different patterns of fQRS in one of our study population.

### Invasive CA

Invasive CA was done using the Judkins technique via femoral access. Patient was considered to have isolated CAE if there was abnormal coronary dilatation segmental or diffuse (but not focal) > 1.5 times the reference normal vessel either in the same artery or in other adjacent normal arteries [1] with no stenosis. Normal coronaries are those without CAE, stenosis, or atherosclerotic irregularities. Based on data from CA, patients were divided into 2 groups (isolated CAE vs. normal coronaries) each containing 50 patients. The 2 groups were compared regarding all study variables to see if there were any significant differences.

### Statistical analysis

Analysis was performed using the Statistical Package for the Social Sciences (SPSS) version 22.0 for Windows (SPSS Inc., Chicago, IL, USA). Categorical variables were described as percentages. Continuous variables were described as mean ± SD. Categorical data were compared using the *χ*^2^ test. Continuous variables were compared between groups using the Student *t* test or Mann-Whitney *U* test. Multivariate logistic regression analysis was used to find independent predictors of CAE. Receiver operating characteristic (ROC) curve was used to evaluate independent risk factors for CAE and their predictive values. *P* values < 0.05 were considered significant.

## Results

### Clinical and demographic characteristics

**Fig. 2:**
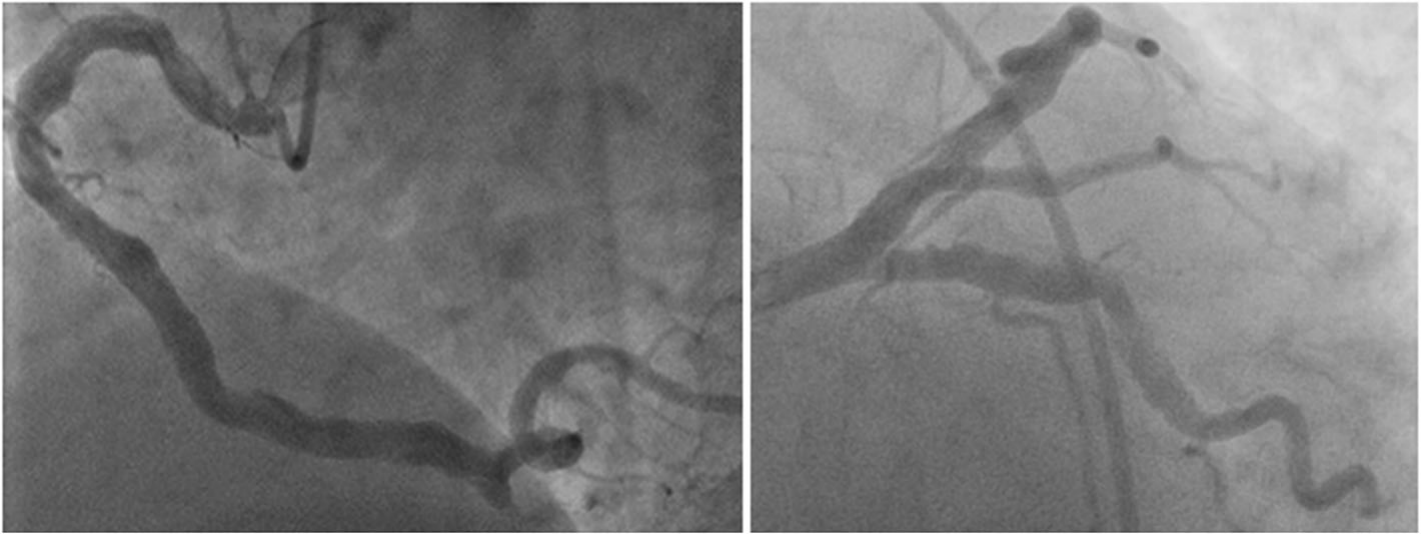
Coronary angiography of one of our study population showing diffuse ectasia of RCA (left), and LAD and LCX (right)

Clinical and demographic data of our study population are shown in Table 1. Isolated systolic HTN was found in most of our hypertensive patients (19 with normal coronaries and 22 with CAE). Coronary ectasia had single vessel distribution in 19 patients versus multivessel distribution in 31 patients. According to Markis et al.’s classification [2], 19 patients had type I, 12 patients had type II, 10 patients had type III, and 9 patients had type IV. Left anterior descending coronary (LAD) was affected in 39 cases, left circumflex coronary (LCX) in 21 cases, right coronary artery (RCA) in 33 cases, and left main (LM) coronary artery in only one case. Figure 2 shows ectasia of RCA, LAD, and LCX in one of our study population. fQRS affected inferior leads in 15 cases, anterolateral leads in 15 cases, anterior leads in 11 cases, all leads in 8 cases, lateral leads in 2 cases, anteroseptal leads in one case, and inferior and high lateral leads in one case. There was no significant difference between the 4 types of CAE as regards the incidence of fQRS.

**Table 1:** Demographic, clinical, ECG, and echocardiographic data of our study population

Variables	Total
Age (years)	55.10 ± 9.19
Male sex	69 (69.0%)
Smoking	43 (43.0%)
Diabetes mellitus	46 (46.0%)
Hypertension	48 (48.0%)
Positive family history of premature IHD	16 (16.0%)
Dyslipidemia	23 (23.0%)
Systolic blood pressure (mmHg)	125.15 ± 15.64
Diastolic blood pressure (mmHg)	79.20 ± 10.88
fQRS	53 (53%)
Pulmonary artery pressure (mmHg)	36.76 ± 14.33
Mitral regurgitation (any grade)	51 (51%)
Diastolic dysfunction (any grade)	44 (44.0%)

### Comparison between the 2 study groups

Univariate logistic regression analysis showed a significant correlation between male sex, smoking, diabetes mellitus (DM), elevated systolic blood pressure (SBP), fQRS, echocardiographic evidence of left ventricular diastolic dysfunction (LVDD), and the incidence of CAE (*P* values of 0.006, 0.003, 0.017, 0.03, 0.0001, and 0.04, respectively). Comparison between both groups regarding different variables is shown in Table 2.

**Table 2:** Comparison between the 2 study groups as regards different study variables

		Normal group (*N* = 50)	Ectasia group (*N* = 50)	*P* value
Age		54.02 ± 9.54 (32–70)	56.31 ± 8.85 (36–72)	0.220
Male sex		28 (56.0%)	41 (82.0%)	**0.005**
Smoking		14 (28.0%)	29 (58.0%)	**0.002**
DM		17 (34.0%)	29 (58.0%)	**0.016**
Dyslipidemia		10 (20.0%)	13 (26.0%)	0.476
Family history of premature IHD		6 (12.0%)	10 (20.0%)	0.275
Hypertension		22 (44.0%)	26 (52.0%)	0.423
SBP		121.70 ± 15.31 (100–160)	128.60 ± 15.35 (100–160)	**0.027**
DBP		78.80 ± 12.06 (60–110)	79.60 ± 9.68 (60–100)	0.715
QRS fragmentation		6 (12.0%)	47 (94.0%)	**0.000**
Pulmonary artery pressure (mmHg)		36.70 ± 14.90	36.82 ± 13.90	0.967
Mitral regurgitation	Grade 1	19 (38%)	18 (36%)	0.663
	Grade 2	6 (12%)	5 (10%)	
	Grade 3	2 (4%)	1 (2%)	
	Grade 4	0 (0%)	0 (0%)	
Diastolic dysfunction	Grade 1	17 (34.0%)	24 (48%)	**0.044**
	Grade 2	0 (0%)	3 (6%)	
	Grade 3	0 (0%)	0 (0%)	
	Grade 4	0 (0%)	0 (0%)	

Multivariate logistic regression analysis showed that QRS fragmentation in surface ECG is the most important independent predictor of CAE (*P* < 0.00001; OR 407.741, 95% CI 35.417–4694.164). The ROC curve was used to assess the accuracy of using QRS fragmentation in the prediction of CAE, and it showed an overall accuracy of 91%, sensitivity of 94%, specificity of 88%, positive predictive value of 88.7%, and negative predictive value of 93.6%.

### Correlation of fQRS distribution on surface ECG and anatomical CAE location

We found a highly significant correlation between increase in the number of ECG territories affected by fQRS and multivessel affection by CAE (*P* = 0.00001). We also found a highly significant correlation between LAD ectasia and fQRS affecting anterior leads [including fragmentation in anterolateral and anteroseptal leads] (*P* = 0.00001). Our results also showed a significant correlation between LCX ectasia and fQRS affecting lateral leads (*P* = 0.003) followed by inferior leads (*P* = 0.04). Also, a highly significant correlation between RCA ectasia and fQRS affecting inferior leads was found (*P* = 0.00001).

## Discussion

Our study was an observational prospective case control study that was conducted upon 2 groups (isolated CAE vs. normal coronaries group) each one consisting of 50 patients based on data obtained from invasive CA. We aimed to find out some clinical characteristics that can non-invasively predict the presence of isolated CAE. Univariate analysis showed that there is a significant correlation between male sex, smoking, DM, elevated SBP, fQRS, echocardiographic evidence of LV diastolic dysfunction, and the incidence of CAE. Multivariate analysis showed that fQRS in surface ECG is the most important independent predictor of CAE.

Due to sparsity of data, there is no agreement about definite risk factors for CAE. However, some studies postulated that the classic risk factors for atherosclerotic CAD may have a considerable role in the development of CAE [24, 29].

Many studies showed that male sex is an independent risk factor for CAE [25, 30, 31]. This gender difference suggests a possible protective role of estrogen against the development of CAE.

A significant correlation between smoking and incidence of CAE was found. Similar data were obtained by Boles et al. in their study [23]. On the other hand, Qin et al. [31] in their study failed to prove such a correlation. Despite the association between smoking and CV disease is well proven with solid evidence, yet the exact pathological details of this link are not yet fully understood [32].

The correlation between DM and CV diseases is unquestionable. It is known from literature that even with proper control of glycated hemoglobin and adjustment of all other CV risk factors, two thirds of patients with type 2 DM are predicted to die suffering from a CV complication [33]. Qin et al. found no significant difference between their study groups regarding incidence of DM [31]. Surprisingly, Bermudez et al. found that absence of DM is an independent predictor for CAE [29]. Diabetic patients on the other side of the spectrum are taking hypoglycemic drugs, statins, and angiotensin blockers which might have a protective effect.

HTN was found to be an independent predictor of CAE in many studies [26, 30, 34, 35]. Kobeissi et al. found in their meta-analysis that hypertensive patients have 66% higher risk for developing abdominal aortic aneurysm (AAA) than normotensive individuals [35], given a nearly similar pathology in both vascular beds [36]; this finding can be extrapolated to coronary arteries. CAE patients in our study had significantly higher SBP than patients with angiographically normal coronaries (a correlation that proved non-significant in multivariate analysis), but we did not find such a significant difference between both groups regarding DBP. Increased SBP with low or normal DBP is linked to arterial stiffness [37] which is postulated as a protection against arterial aneurysmal dilatation [38], and this can explain the non-significant effect of HTN in our study given that most of our hypertensive patients in both groups had isolated systolic HTN. Also, the correlation between echocardiographic evidence of LVDD and CAE may be indirect via other factors that cause DD as HTN and myocardial ischemia.

Multivariate logistic regression analysis of our study data showed that QRS fragmentation in 12-lead surface ECG is the most important independent predictor of CAE. To our knowledge, the study done by Sen et al. was the only study that discussed the correlation between fQRS and isolated CAE. They found that fQRS is significantly more found in patients with isolated CAE than in patients with normal coronaries [39]. fQRS is more frequently seen in patients with slow coronary flow which is an indicator of microvascular dysfunction [40]. This association between fQRS and coronary slow flow was discussed in some studies as that was done by Mittal [41]. Microinfarctions caused by CAE combined with microvascular dysfunction can be a possible cause for abnormalities in myocardial depolarization resulting in this ECG abnormality. The extremely high incidence of fQRS in our ectasia group might be related to delayed presentation due to decreased medical awareness leading to more subclinical ischemia and microinfarctions. This assumption needs further confirmation in future studies with larger numbers of patients.

To our knowledge, our study is the first one that elucidates the significant correlation between fQRS distribution in surface ECG and anatomical distribution of CAE. Our results in this aspect can make us move a further step beyond just using fQRS as a predictor of CAE towards predicting which artery is affected. These correlations after being confirmed in larger studies can definitely be of utmost importance in more accurate risk stratification of this category of patients.

Our study limitations were the small sample size, and future studies with larger sample sizes will be needed to confirm our results with homogenous distribution of classic cardiovascular risk factors between both groups and allow comparison of fQRS patterns in different types of CAE. Also, the study was done in a single center. We did not use any non-invasive imaging modalities to confirm the association between CAE and scar in the same territory. All our patients were only Caucasians. A larger number of patients are needed to evaluate the separate effect of fQRS in each territory of surface ECG and compare their relative risk, similarly to assess the effect of single vessel ectasia versus multivessel ectasia and the separate risk for affection of each coronary territory. Patients presenting with acute coronary syndromes can be included in future studies. Also, patients with combined ectasia and stenosis need to be studied.

## Conclusion

In conclusion, our study showed that fQRS is an independent predictor for the presence of isolated CAE and can be used to predict its anatomical distribution. Our results if proved in other studies with larger number of patients can give data for interventional cardiologists about the possibility of facing that challenging abnormality in their patients before going to the cath lab via a simple non-invasive predictor and can also make them anticipate anatomical distribution of CAE using the territorial affection in surface ECG. Finding such a method to anticipate this situation is of utmost importance especially in cases of emergencies if other studies including patients with unstable CAD showed findings similar to ours as it gives an alarm regarding more possible complications and the need for more aggressive anticoagulation approach that may even be started before the procedure after doing the surface ECG. Such finding during routine checkup can also be a drive to start employing intensive risk modification strategies in those patients even before suffering from acute coronary syndromes.

## Data Availability

The datasets used and analyzed during the current study are available from the corresponding author on reasonable request.

## Abbreviations

ABP: Arterial blood pressure
CA: Coronary angiography
CAD: Coronary artery disease
CAE: Coronary artery ectasia
DBP: Diastolic blood pressure
DM: Diabetes mellitus
fQRS: Fragmented QRS in 12-lead surface ECG
HTN: Hypertension
LAD: Left anterior descending coronary
LCX: Left circumflex coronary
LVDD: Left ventricular diastolic dysfunction
LVH: Left ventricular hypertrophy
MI: Myocardial infarction
RCA: Right coronary artery
SBP: Systolic blood pressure
